# Dysfunction of insulin-AKT-UCP1 signalling inhibits transdifferentiation of human and mouse white preadipocytes into brown-like adipocytes

**DOI:** 10.1080/21623945.2022.2062852

**Published:** 2022-04-13

**Authors:** Jie Pan, Suchart Kothan, Aye Thidar Moe Moe, Kun Huang

**Affiliations:** a Shandong Provincial Key Laboratory of Animal Resistant Biology, College of Life Sciences, Shandong Normal University, Jinan, Shandong Province, China; b Center of Radiation Research and Medical Imaging, Department of Radiologic Technology, Faculty of Associated Medical Sciences, Chiang Mai University, Chiang Mai, Thailand

**Keywords:** Insulin signalling, AKT, UCP1, white preadipocytes, browning

## Abstract

The mechanism of insulin signaling on browning of white preadipocytes remains unclear. Human and mouse primary subcutaneous white preadipocytes (hsASCs and WT lean and obese msASCs, respectively) were induced to transdifferentiate into beige adipocytes under conditions of intact or blocked insulin signaling, respectively. Level of phosphoinositide-3-kinase (PI3K) after induction of beige adipocytes under conditions of normal insulin signaling, phosphorylated protein kinase B (pAKT), peroxisome proliferator-activated receptor γ coactivator-1 alpha (PGC-1α), zinc-fifinger transcriptional factor PRD1-BF1-RIZ1 homologous domain-containing protein 16 (PRDM16), uncoupling protein 1 (UCP1), peroxisome proliferator-activated receptor gamma (PPARγ) and CCAAT/enhancer binding protein beta (C/EBPβ) were significantly increased. Conversely, when insulin signaling is incompletely inhibited, the expression of the thermogenic and adipogenic factors is significantly reduced, with obvious impairment of adipogenesis. However, phosphorylation level of adenosine 5’-monophosphate (AMP)-activated protein kinase (AMPK) and expression level of sirtuin type 1 (SIRT1) had increased. These white preadipocytes from different donors showed similar dynamic change in morphology and molecular levels during the browning. The present data indicate that insulin signaling plays a important role in regulation of browning of hsASCs and msASCs through PI3K-AKT-UCP1 signaling pathway. The insulin-AMPK-SIRT1 pathway was also involved in the adipocytes browning, while its effect is limited.

## Introduction

Brown adipocytes (BACs) consumes energy in the form of heat production and is used as a defence mechanism against hypothermia, especially in infants, and it also contributes to metabolic homoeostasis [1–5], that protects against obesity [6–10]. Starting in neonates, the white adipose tissue (WAT) rapid proliferates, with age. Conversely, the absolute amount of brown adipose tissue (BAT) declines rapidly with age. When compared with WAT that accounts for approximately 20% of body weight in healthy adults, BAT is only approximately 50 grams [3]. Recently, inducible brown-like (beige) adipocytes were found in WAT and BAT [4–6]. Activation of thermogenic molecules, including mitochondrial uncoupling protein 1 (UCP1) and lipolysis, was considered as a kind of potential therapeutic strategy to combat obesity [4,7–9]. It has been found that β-adrenergic stimulation and cold exposure not only can induce BACs activation [10–14] but also white adipocytes (WACs) browning [5,8–11]. These beige cells appear to have characteristics between BACs and WACs in cell morphology and cell biology. Activated beige cells also can be involved in thermogenesis, and can transform into brown adipocytes by certain stimuli [5,12]. In addition, cold acclimation activates the β-adrenergic receptor pathways through sympathetic nerves and increases the formation of BACs [5,11,13,14], thereby increasing insulin receptor substrate, extracellular regulatory protein kinase, and protein kinase B (AKT) phosphorylation levels [14]. Deletion of adipocyte AKT causes severe WAT atrophy, and was accompanied by insulin resistance and other metabolic syndrome phenotypes [15]. Our previous study showed that insulin signalling negatively regulates humans and mice WACs dedifferentiation through the PI3K-AKT-mTORC1 signalling pathway [16,17].

These phenomena may suggest that intact insulin signalling not only regulates the adipogenesis of white preadipocytes and maintains mature WACs phenotype [17], but also participates in the regulation of WACs browning. It has been reported that the thermogenesis induction of adipose tissues was not strictly dependent on insulin/insulin-like growth factor-1 (IGF-1) signalling [18], while clinical studies have shown that an insulin sensitizers known as glitazones, can increase BAT activity and induce browning of white adipocytes [19], suggesting that insulin responsiveness and browning are connected. However, the underlying mechanism of insulin signalling regulating white preadipocyte transdifferentiation into beige adipocytes has not been thoroughly investigated. We hypothesize that the insulin-AKT signalling pathway is one of the major regulatory networks involved in the browning of WACs. In order to study the effects of insulin signalling on beige adipogenesis of human and mouse white primary preadipocytes, and whether effects of insulin/insulin signalling are species (human and mouse) and mouse phenotypes (lean and obese) independent, we comparetively analysed characteristics of browning in these preadipocytes under conditions of intact and hypofunction of insulin signalling. The relationship of regular adipogenic differentiation (such as lipid accumulation), browning and insulin signalling pathway-related molecules are being studied.

## Materials and methods

### Chemicals s and reagents

Insulin, dexamethasone (Dex), 3-isobutyl-1-methylxanthine (IBMX), collagenase type I, and linsitinib (OSI-906) were purchased from Sigma-Aldrich (USA). Dimethyl Sulphoxide (DMSO) was purchased from Merck (Darmstadt, Germany). TRizol, Dulbecco’s modified Eagle’s-F12 medium (DMEM/F12), foetal bovine serum (FBS), and an antibiotic mixture (penicillin- streptomycin) were purchased from GIBCO-BRL (Grand Island, NY).

### Human primary preadipocytes and culture

The cells used in this study were primary human subcutaneous preadipocytes (hsASCs) cryopreserved in our laboratory. They were isolated from subcutaneous WAT of plastic surgery healthy recipients, and were digested in 0.1% (v/v) collagenase type I solution (containing 0.4% BSA, v/v), at 37°C water bath with constant agitation at 100 rpm for 60 minutes. The cell pellets were then resuspended in a cell growth medium (CGM, DMEM/F12 containing 10% FBS (v/v), 100 U/ml penicillin and 100 µg/ml streptomycin), filtered through a 100 µm strainer, and were seeded into 25 cm^2^ flasks, called F0 cells. Based on the fact that mesenchymal stromal cells (MSCs) takes longer to attach to the bottom of a dish than fibroblasts and are more sensitive to trypsin, a strategy of differential adherence and incomplete digestion is used to gradually improve the purity of MSCs. Briefly, when the F0 cells grow to 70% confluence, the old culture medium is disgarded and the cells are rinsed with DMEM/F12, followed by adding 1 ml 1% of trypsin to digest the cells at 37C for 1–2 minutes. After 50% of the cells start to float, 2 ml of CGM is gently added, cell suspension is collected, and cell pellets are collected after centrifugation for 1 minute at 800 rpm. The cells were reseeded into 25 cm^2^ flask, and labelled as F1 generation. When F1 cells were confluent to 70%, the purification steps were repeated as described above. After final purification the F4 or F5 cells were seeded onto 12-well dishes. These cells were used to induce differentiation to brown-like adipocytes.

### Isolation of mouse primary preadipocytes and culture

Leptin receptor spontaneous heterogeneous point mutate (Lepr*^db/+^, db/+*) C57BL mice were purchased from the Jackson Laboratory (Bar Harbour, USA), and were inbred to obtain homogeneous Lepr*^db/db^* (*db/db*) mice. Genotypes of these mice were identified by PCR. In view of spontaneous obesity in *db/db* mice over 4 weeks of age, the genotype can also be confirmed by obese phenotype. C57BL wild type (WT) mice were purchased from Shanghai SLAC Laboratory Animal Co., Ltd (Shanghai, China) as a lean control. All mice were kept and bred in a temperature-controlled room (22 ± 2°C) on a 12:12-hour light–dark cycle in specific pathogen-free animal facilities with a standard diet and water ad libitum from 22 d of age. Each time, six to eight week-old two genotypes of male mice (n ≥ 11, for each group) were used to isolate subcutaneous primary preadipocytes for experiments, using a collagenase type I digestion method that has been described earlier, while digestion time was shortened to 30 minutes. Mouse adipose-derived stem cells (ASCs) were also purified by differential adhesion and an incomplete digestion method similar to the isolation and purification of hsASCs. The WT and obese mice subcutaneous preadipocytes were named as WT msASCs and obese msASCs, respectively.

### Induce hsASCs and msASCs browning to beige adipocytes

**Figure 1.**
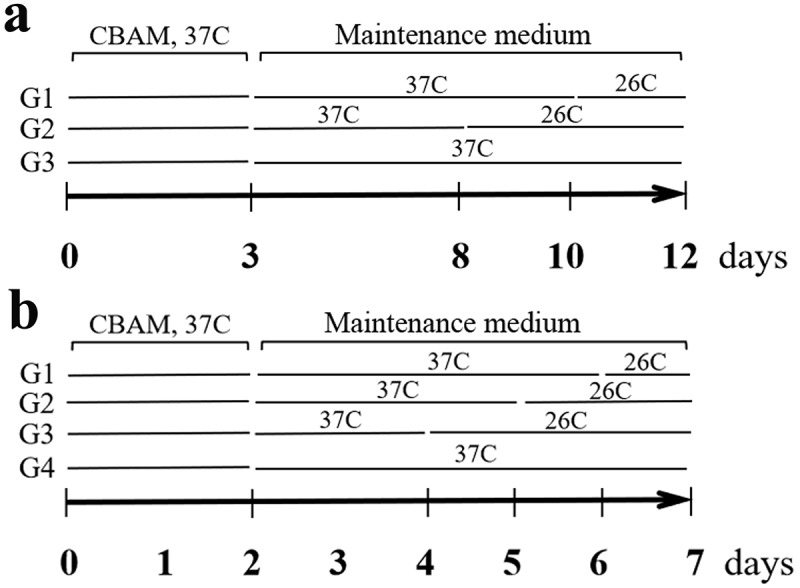
Time course of cell grouping and treatment of low temperature. (a), browning inducing for hsASCs, and (b), msASCs under conditions of different temperature treatment. CBAM, complete browning adipogenic medium. Maintenance medium, as adipogenic medium, for sustained induction of the cell browning. G, group. See the methods in the text for details.

The third-generation of hsASCs were placed into 12-well plates. After the cells were 100% confluent for 48 hours, the medium was replaced with a complete beige adipogenic medium (CBAM, CGM supplemented with 66 μM insulin, 250 mM IBMX, 100 μM Dex, 1 mM rosiglitazone (Rosi), 1 μM T3, 250 μM indomycin and 10 mg/mL transferin, from Sigma-Aldrich, USA). The date was recorded as differentiation day 0 (D0). Three days later, the medium was replaced with a differentiation maintain medium (CGM with 66 μM insulin, 1 mM Rosi, 1 μM triiodothyronine (T3) and 10 mg/mL transferrin, from Sigma-Aldrich, USA) for 9 days. Moreover, to investigate if low-temperature exposure influences beige adipocyte formation, part of the hsASCs were exposed at 26°C from D8 or D10 for 4 or 2 d, respectively, until D12 (Figure 1(a)). The medium was changed every 2–3 d. For msASCs browning induction, fourth-generation of the cells in density of 2 × 10^4^ cells/cm^2^ were cultured in 12-well plates. After the cells were post-confluent (D0), they were exposed to CBAM (CGM supplemented with 17 nM insulin, 1 µM Dex, 0.25 mM mM IBMX, 1 mM Rosi and 1 μM T3), and the date was recorded as D0. Two days later, the medium was replaced with a beige adipogenic maintenance medium (CGM supplemented with 17 nM insulin, 1 mM Rosi and 1 μM T3), and were further cultured for 5 d. Moreover, parts of the WT msASCs were exposed at 26°C from D6 for 1 d, from D5 for 2 d, and from D4 for 3 d in the beige adipogenic media (Figure 1(b)).

### Incompletely blocked insulin signalling in hsASCs and msASCs

The hsASCs and msASCs were set up into two groups. One group was induced for beige adipogenesis without inhibition of insulin signalling at 37°C. Another group was induced for beige adipogenesis accompanied with different concentrations of OSI-906 from D0 to incompletely block both insulin and insulin-like growth factor 1 (IGF-1) signallings.

### Lipid accumulation in beige adipocytes and quantified analysis

Lipid formation in beige adipocyte was identified by Nile red staining. To quantify intracellular lipid accumulation, the cells were fixed with formalin and were stained with Oil Red O (ORO) solution (60% saturated in isopropanol). Intracellular ORO was extracted using isopropanol, and light absorbance by the extracted dye was measured (optical density, OD at 520 nm). The content of samples at several time points (*n* ≥ 7) was determined according to the Lowry method, the data was indicated as an adipogenic differentiation ratio using percentages of appropriate Ctrl (+).

### RT-qPCR

Total cellular RNA was isolated from cells at various time points in each group as previously described [16]. The primers used in this study are shown in Supplementary Table S1. The amount of transcript was normalized to internal α-tubulin and was averaged from triplicate samples.

### Western blot analysis

Cellular proteins were isolated from cells at various time points for each group, and immunoblotting was performed as previously described [17]. The sources of primary antibodies used in this study are as follows: UCP1 (Abnova, USA), PGC-1α, AMPK, pAMPK, PPARγ (Cell Signalling Technology), AKT (Abcam, USA), phosphorylated AKT (pAKT, Thr308, Bioworld, USA), PRDM16, SIRT1 and C/EBPβ (Santa Cruz Biotechnology, USA). Protein levels were normalized to α-tubulin (GenScrept, USA).

### Statistics

All data were presented as mean ± SEM. Two-way ANOVA followed by Tukey’s Multiple Comparison Test was used to compare more than two groups. All experiments were repeated 5–7 times. The differences for the experiments were considered statistically significant at a *p* < 0.05.

## Results

### Incompletely blocking insulin signalling can inhibit hsASCs browning

**Figure 2.**
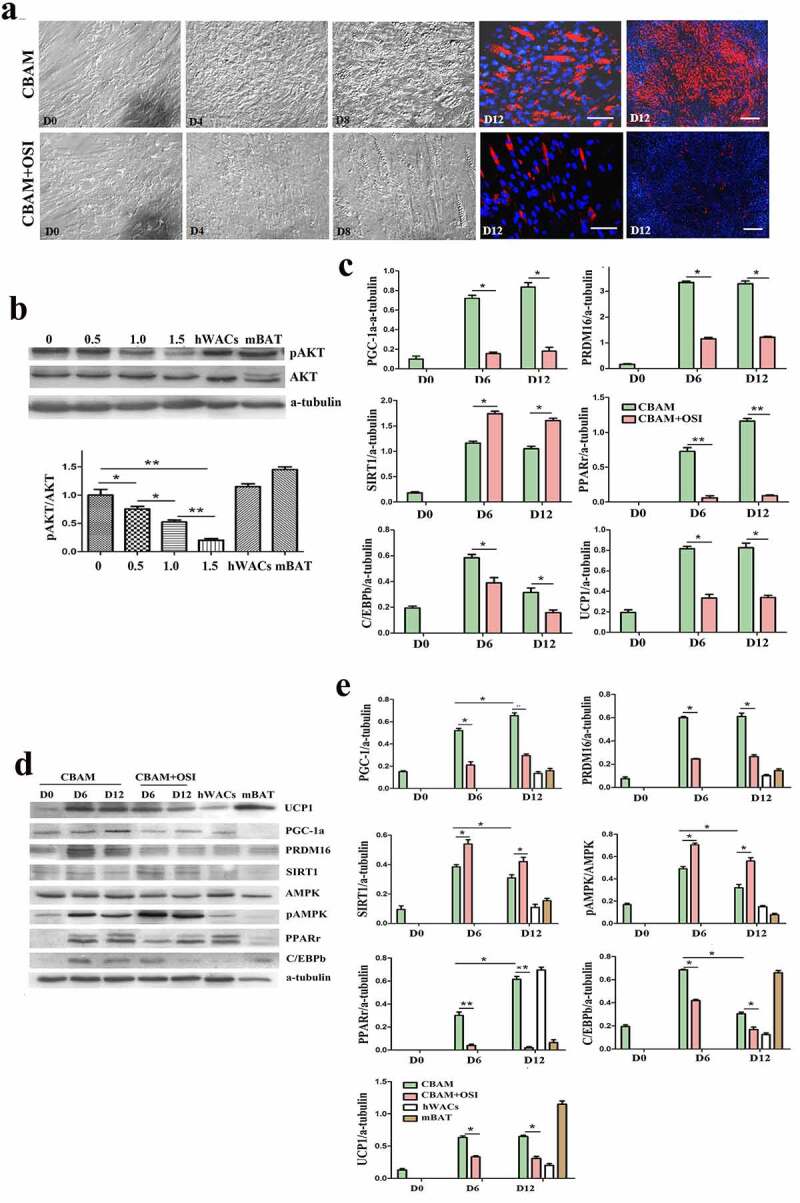
Dynamic changes of primary hsASCs during browning under different conditions. (a), Dynamic changes in cell morphology. CBAM, cells were induced to browning with complete induction medium at 37°C. CBAM+OSI, treatment with 1 μM OSI-906 while inducing browning, inhibits insulin signalling throughout browning induction. Notably, OSI-906-treated cells exhibited significantly suppressed adipogenesis compared to CBAM-induced cells, i.e. from 72 ± 3.5% in the CBAM group down to 7 ± 1.12%. Red, Nile red staining of lipids. Blue, Hoechst 33,342 nuclear staining. Dn, days of induction. b, Western blot and quantitative protein expression analysis of key molecules in insulin signalling, showing that pAKT is decreased in an OSI-906 dose-dependent manner. Numbers marked above the protein bands represent the final concentration (μM) of OSI-906. hWACs, human white mature adipocytes; mBAT, WT mouse BAT, protein samples used as non-intervention controls for analysis. c, Target gene expression profiles analysed using qPCR, and (d), Western blot analysis of target proteins of browning-related molecules in hsASCs under conditions that intact normal or partially inhibit insulin signalling. After insulin signalling was blocked (CBAM+OSI), adipogenic molecules were significantly downregulated and browning markers were significantly reduced, but SIRT1 was significantly increased compared to cells with intact insulin signalling (CBAM). (e), Quantitative analysis of target protein expression. Compared with the qPCR data, the target molecule expression levels showed a similar dynamic trend. However, in contrast to the down-regulation of the expression levels of these molecules, pAMPK levels and SIRT1 expression were instead increased after insulin signalling was inhibited. *p < 0,05 and **p < 0.01 represent the different levels between groups (*n* > 7).

Judging from cell morphology and the expression characteristics of detected molecules, we confirmed that the hsASCs were differentiated into beige adipocytes after induction of browning at 37°C using our method. Morphologically, the cells appeared small lipid droplets 3 da after induction (Figure 2(a), CBAM), and the number of lipid droplets gradually increased with the extension of induction time. In D12, the percentage of adipogenesis was about 72 ± 3.50%. These beige cells with small lipid droplets and the biological characteristics that distinguish them from adipogenic differentiated WACs was induced by using a typical MDI method [16]. However, in the insulin signalling inhibition group, adipogenesis was reduced to 7 ± 1.12% (Figure 2(a), CBAM+OSI). A key molecule of insulin signal pathway, AKT was not significantly changed in total protein level, while the level of pAKT was reduced by 87 ± 2.49%. This result indicates that insulin-AKT pathway was mostly inhibited, but it was not blocked completely. The degree of pAKT decline is OSI-906 dose-dependent (Figure 2(b)). As shown in Figure 2(c-e), expression of UCP1, PGC-1, PRDM16, PPARγ,+ and C/EBPβ, both in mRNA and protein levels, were all dramatically down-regulated after inhibition of insulin signalling compared to control cells. These results paralleled with the dynamic changes in cell morphology, i.e. less lipids accumulation and/or fibroblast-like shape which was undifferentiated. In the following experiments, we selected 1 μM of OSI-906 at a final concentration to treat the cells in order to partially inhibit insulin signalling, because having a reserve of a certain level of insulin signalling is indispensable for cell survival.

### Incompletely blocking insulin signalling inhibits msASCs browning

**Figure 3.**
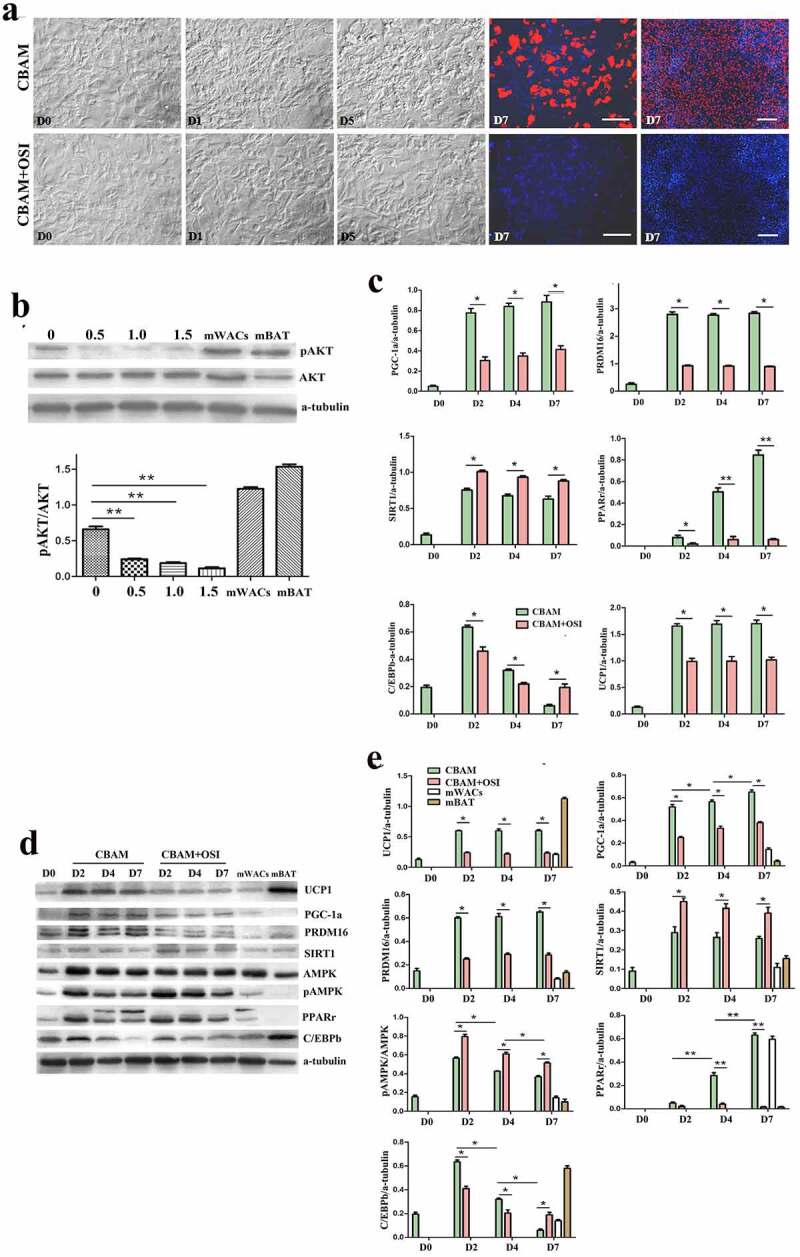
Browning characteristics of WT msASCs under intact and insulin signalling inhibited situations. (a), dynamic changes of cell morphology. Compared to the cells induced by CBAM, OSI-906 treated cells (CBAM+OSI) showed significantly repressed adipogenesis, from 78 ± 4.5% down to 5 ± 1.5%. Red colour, Nile red staining for lipids. Blue colour, nuclear staining by Hoechst 33,343. Dn, the number of days of induction. (b), Western blotting and protein expression quantification analysis of pAKT, the level of pAKT is decreases in OSI-906 dose dependent manner. Numbers labelled above the protein bands represent final concentration (μM) of OSI-906. The degree of insulin signalling inhibition depended on the dosage of OSI-906. mWACs, WT mouse white mature adipocytes; mBAT, WT mouse BAT, used as controls. (c, d and e), Target gene and protein expression of browning and adipogenic molecules in WT msASCs under conditions of intact and incomplete inhibition of insulin signalling were analysed by qPCR and Western blotting. Beige adipogenesis and browning marker molecules were significantly downregulated after insulin signalling was inhibited (CBAM+OSI), while SIRT1 was significantly increased compared to insulin signalling intact cells (CBAM). In contrast to the down-regulation of these molecules, pAMPK levels and SIRT1 expression instead increased after insulin signalling was inhibited. The dynamic trends of target gene mRNA and protein levels were similar to those seen in hsASCs. **p < 0.01 represent the different levels between groups (*n* > 7).

**Figure 4.**
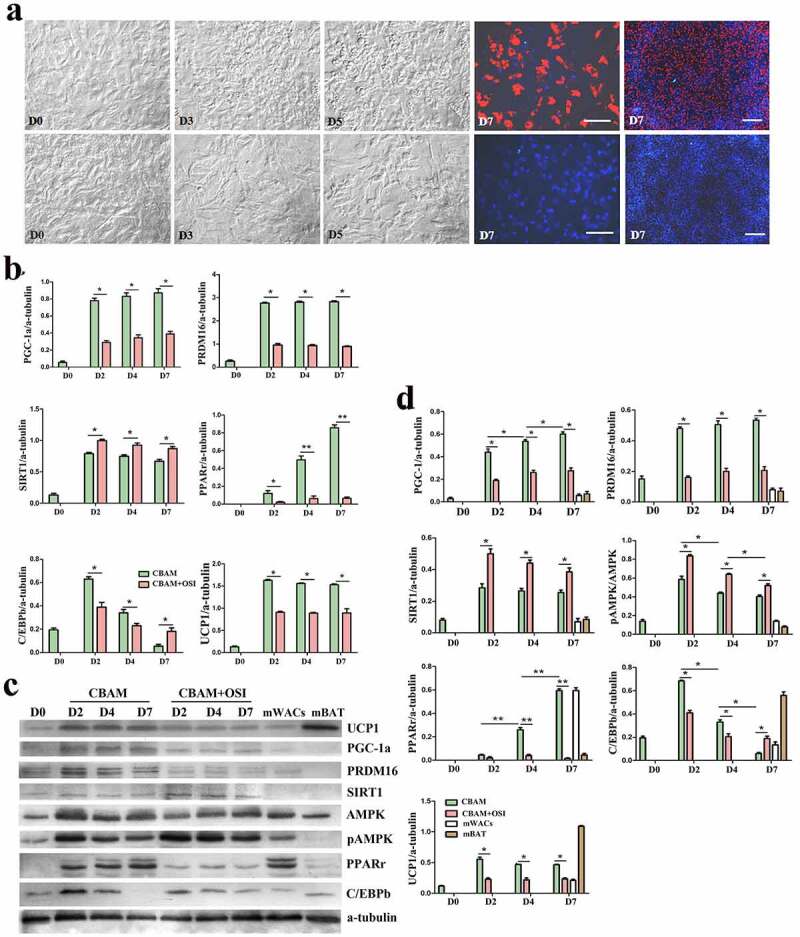
Dynamic changes of obese *db/db* msASCs in browning under different conditions. a, morphology of the cells. CBAM, the cells were induced for browning by using a complete inducing medium under 37°C. CBAM+OSI, the msASCs were induced for browning and also treatment the cells with 1 μM OSI-906, showing lipids production and accumulation of the cells was significantly inhibited. Red colour, Nile red staining for lipids. Blue colour, nuclear staining by Hoechst 33,342. Dn, the number of days of induction. b, qPCR analysis of target genes. c, Western blotting, and d, quantification of protein expression of *db/db* msASCs during browning. mWACs, WT mouse subcutaneous white mature adipocytes. mBAT, WT mouse BAT, as UCP1 positive control, its total protein was loaded 1/3 compared to other samples. Adipogenesis decreased significantly after inhibition of insulin signalling. On the contrary to down-regulation of insulin signalling molecules and browning markers, pAMPK level and SIRT1 expression were increased after insulin signalling was inhibited. These results were similar as those seen both in WT msASCs and hsASCs. *p < 0.05 and **p < 0.01 represent the different levels between groups (*n* > 7).

In order to confirm whether the effect of insulin/insulin signalling on browning of white preadipocytes is independent of mouse genotype and phenotype, we compared the differences in cell morphology, and expression patterns of target molecules of msASCs in two phenotypes of mice (lean WT and obese *db/db*) under insulin signalling inhibition and intact state. We found that adipogenesis rate had decreased from 78 ± 4.50% to 5 ± 1.36% after inhibition of insulin signalling in WT msASCs (Figure 3(a), CBAM vs CBAM+OSI). Compared to the insulin signalling intact group, pAKT level was also reduced in an OSI-906 dose-dependent fashion (Figure 3(b)), which is a similar tendency that is seen in hsASCs. After incomplete inhibition of insulin signalling, pAKT decreased by 91 ± 1.23%. After insulin signalling was incompletely inhibited, the WT msASCs also showed significant decrease in expression of brown adipocyte markers and adipogenic factors, and there was a significant suppression of adipogenesis (Figure 3(c-e)). Obese msASCs also can be induced into beige adipocytes under intact insulin signalling, while the differentiation rate was 58 ± 3.27% (Figure 4(a), CBAM) which was slightly lower than that of WT msASCs. Moreover, the differentiation was reduced to 4 ± 1.14% when the insulin signalling was inhibited (Figure 4(a), CBAM+OSI). In addition, the expression of PGC-1, PRDM16, PPARγ, C/EBPβ, and UCP1, both in mRNA (Figure 4(b)) and protein (Figure 4(c) and 4(d)) levels were also significant down-regulated when the insulin signalling was inhibited.

### Inhibition of insulin signalling leads to a compensatory increase of SIRT1 and pAMPK

It should be noted that when the inhibition rates of insulin signalling in hsASCs and msASCs were 87 ± 2.49% and 91 ± 1.23%, respectively, other browning related molecules such as SIRT1 and pAMPK were significantly increased as shown in Figure 2(c-e) (hsASCs), Figure 3(c-e) (WT msASCs) and Figure 4(b-d) (obese msASCs). This increase may be due to a compensatory response mechanism after suppression of the insulin-PI3K-AKT signalling pathway, suggesting that they also participate in the formation of beige adipocytes.

### Low temperature exposure reduces lipid accumulation, but promotes browning

**Figure 5.**
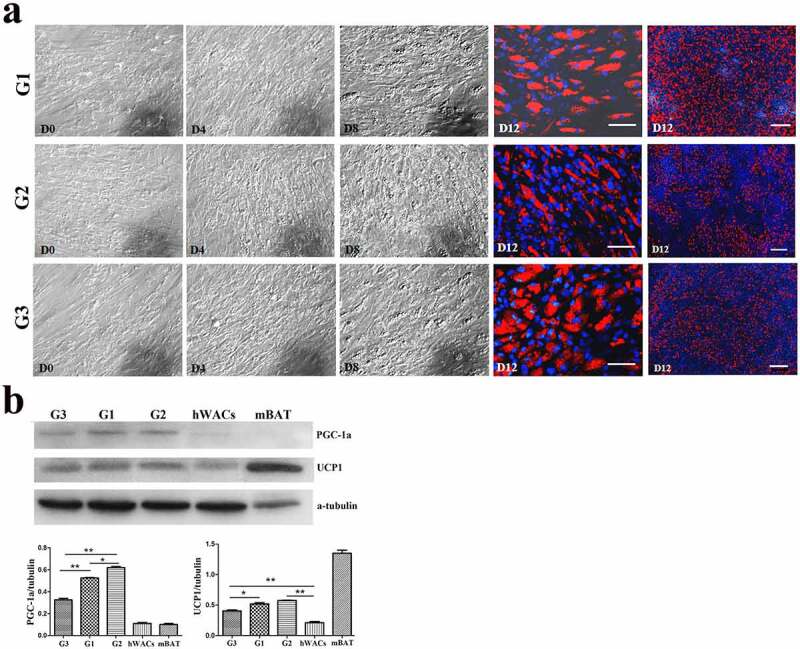
The dynamic changes of the browning of hsASCs which were exposed at 26°C for different days (see the grouping in Figure 1a). (a), dynamic changes of the cell morphology. G1, group 1, browning induction, and the culture temperature was switched from 37°C to 26°C at D10, for 2 d. G2, group 2, browning induction, and the culture temperature was switched from 37°C to 26°C at D8, for 4 d. G3, group 3, browning induction under 37°C for 12 d. Red colour, Nile red staining for lipids. Blue colour, nuclear staining by Hoechst 33,342. Dn, the number of days of induction. (b), Western blotting and quantification of PGC-1α, and UCP-1 proteins expression of different groups of the cells during browning. Adipogenesis decreased upon the period of low temperature treatment (a, G3> G1> G2), while the tendency of browning of the cells was positively correlated with the period of low temperature treatment (b, G2> G1> G3). hWACs, human subcutaneous mature white adipocytes. mBAT, WT mouse BAT, as UCP1 positive control, its total protein was loaded 1/3 compared to other samples. * *p* < 0.05 and ** *p* < 0.01 represent the different level between groups (*n* > 7).

**Figure 6.**
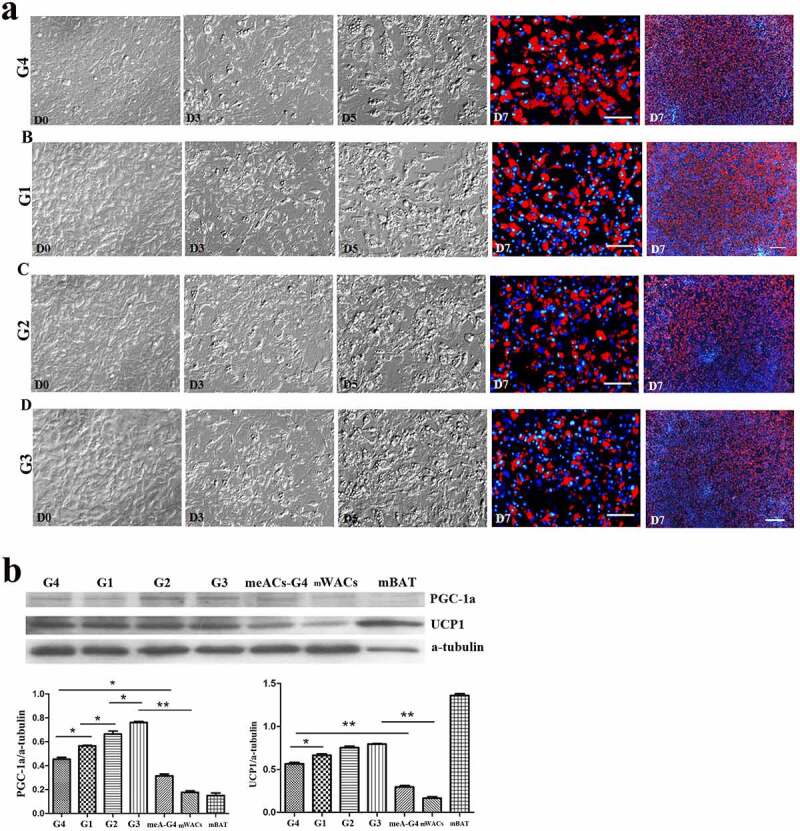
The dynamic changes of the browning of msASCs exposure at 26°C for different days. (a), dynamic changes of the cell morphology. G1, group 1, browning induction, and the culture temperature was switched from 37°C to 26°C at D6, for 1 da. G2, group 2, browning induction, and the culture temperature was switched from 37°C to 26°C at D5, for 2 d. G3, group 3, browning induction, and the culture temperature was switched from 37°C to 26°C at D4, for 3 d. G4, group 4, browning induction under 37°C for 7 d. Adipogenesis decreased during the period of low temperature treatment, and the tendency of induction of cell browning was positively correlated with the days of low temperature treatment, the tendency which was similar as seen in the hsASCs (G3> G2> G1> G4). Red colour, Nile red staining for lipids. Blue colour, nuclear staining by Hoechst 33,342. Dn, the number of days of induction. (b), Western blotting and quantification of PGC-1α and UCP-1 expression during browning. meACs-G4, WT mouse epididymal ASCs, browning under 37°C for 7 d. mWACs, WT mouse subcutaneous white mature adipocytes. mBAT, WT mouse BAT, as UCP1 positive control, and its total protein was loaded 1/3 compared to other samples. * *p* < 0.05 and ** *p* < 0.01 represent the different levels between groups (*n* > 7).

To verify the effect of low temperature on browning of hsASCs and WT msASCs, we comparatively analysed the differences in cell morphology, and expression patterns of browning markers under 37°C and 26°C situations during browning. Low temperature exposure reduced the rate of adipogenesis, i.e. smaller lipid droplets and less cells of beige differentiation. This tendency of dynamic change was positively correlated with the number of days of low-temperature exposure in the hsASCs (Figure 5(a)) and the WT msASCs (Figure 6(a)). For example, when the cells were treated at 26°C from D8 for 4 d, the adipogenesis rate was only 50 ± 2.63% in hsASCs (Figure 5(a), G2). With regard to WT msASCs differentiation into beige adipocytes, they were shown to exhibit a similar tendency of dynamic changes to hsASCs, i.e. the differentiation rate was 60 ± 3.70% (Figure 6(a), G3) at 26°C from D4 for 4 d was slightly higher than that of hsASCs. Notably, compared with reduced adipogenesis that occurred with the extension of the low-temperature exposure period, the browning markers UCP1 and PGC-1α both in the hsASCs (Figure 5(b)) and in the WT msASCs (Figure 6(b)) were significant up regulated. These results indicate that cold acclimation reduces lipids production and accumulation in hsASCs and WT msASCs, but promotes their browning.

## Discussion

The formation of both WACs and BACs is a physiological phenomenon in the body that induces differentiation of preadipocytes into their corresponding mature adipocytes [3,4,8,10]. Based on the similar characteristics in morphology and biological functions of these two types of adipocytes, and even particular respective specific inducers that are required in BACs differentiation, common regulatory signals and transcription regulators such as insulin, PPARs, C/EBPs, and SREBP-1C are critical for both processes [3,7,20–22]. Such different directions of differentiation are the results of the combined influence of different microenvironments that are composed of internal and external signals. Being different than white adipogenic induction in terms of browning of white adipocytes, in addition to typically being adipogenic inducers, other stimuli such as T3, noradrenalin, and cold exposure are also required to induce the expression of browning molecules, such as UCP1, PRDM16, and PGC-1α [7,12,14,20,21], so that different signal microenvironments can be formed that selectively switch to and promote white preadipocyte browning [4,8,18].

Insulin through its receptor signal promotes lipids, carbohydrates, and proteins into macromolecules that are involved in inducing nutrient storage in the body, cell growth, and cell differentiation [14,17,18,22]. Based on the clues of the role of insulin/insulin signalling in the differentiation of preadipocytes and dedifferentiation of mature adipocytes [16,17,19,22,23], we speculate that the insulin-PI3K-AKT signalling network influences the WACs browning by regulating the expression and its function of brown adipocyte markers. In this study, when induced hsASCs and msASCs to differentiate into beige adipocytes that is accompanied with insulin signalling inhibition, while expression of adipogenic factors were obviously down regulated, so that there is impaired lipid accumulation in the cells. Our results confirmed that the insulin signalling, like its role in white preadipocyte differentiation, also positively regulates beige adipogenesis of human and mouse preadipocytes via certain signalling pathways.

Evidence indicates that irisin, PPARγ, myostatin, FGF21, cold temperatures, and β-epinephrine agonist are involved in white adipocyte browning [11,14,19,20,24]. WACs can be converted into beige cells by activating cAMP-PKA/p38 MAPK, AMPK-SIRT1, and PRDM-16 signal pathways and related regulatory factors in WACs [25–29]. PPARγ combines with PRDM16, PGC-1α, and other molecules to form transcription complexes, promotes UCP1 and PGC-1α transcription levels [30–32], and triggers browning. Recent studies have shown that the development and maintenance of BAT in mice requires the participation of AKT, which activates PPARγ, and stimulates browning of adipocytes through a series of cascade reactions [12,15,33]. Inhibiting the PI3K-AKT signalling pathway can significantly reduce the production of WAT in mice and lowers the expression of related adipogenic genes [15–17,34]. However, the effect of insulin signalling on human and mouse subcutaneous preadipocyte browning and related mechanisms have been investigated less extensively. In the present study, after inhibition of insulin signalling in hsASCs and msASCs, the level of pAKT was markedly decreased and was accompanied with significant downregulation of PPARγ and other adipogenic factors in ways similar to phenomenon in mouse white preadipocytes during differentiation into white mature adipocytes [16].

**Figure 7.**
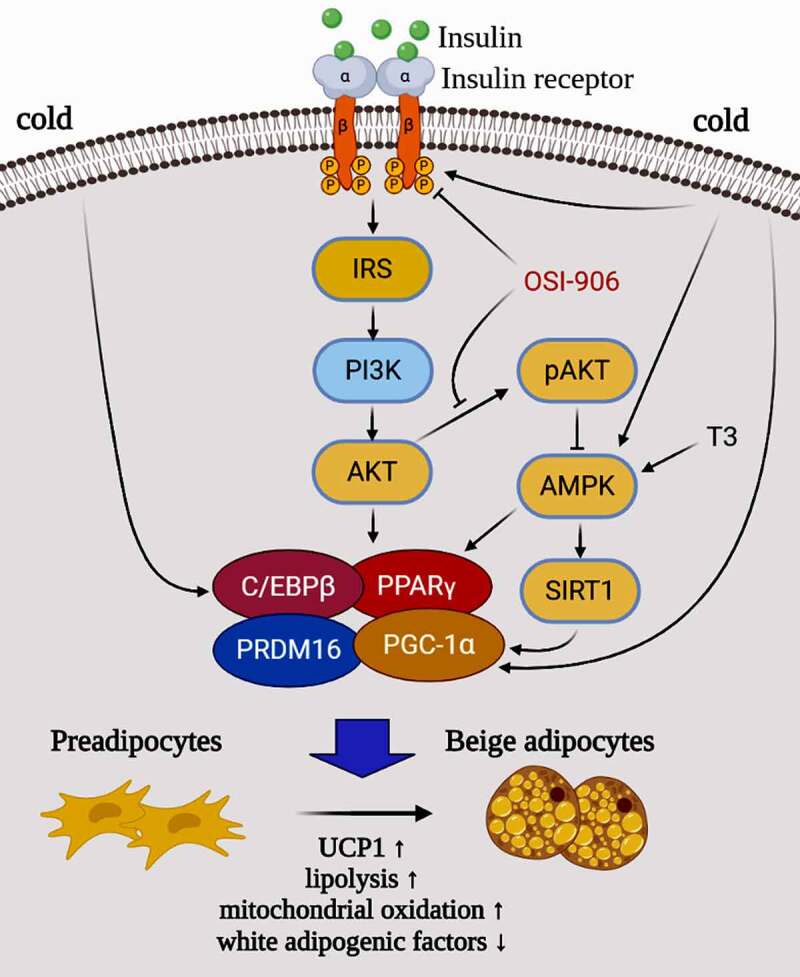
A hypothetical model and potential mechanism of the insulin-PI3K-AKT-UCP1 pathway in animal white preadipocyte browning under different conditions. When insulin signalling is intact, white preadipocytes can be induced into beige adipocytes by the stimuli (such as CBAM in this study) including insulin and T3. However, when OSI-906 was used to block the insulin signal incompeletely, both lipogenesis and Browning were inhibited. Cold exposure induces browning of white adipocytes by affecting the expression and function of C/EBPβ and PGC-1α which are downstream molecules of insulin signalling, and/or molecules (such as AMPK and SIRT1) in a signalling pathway crosstalk with insulin signalling. This indicates that the browning of WAC caused by cold exposure may also related to insulin signalling.

Pharmacological and genetic interventions of PRDM16 in mature adipocytes lower the phosphorylation level of AKT, while PPARγ agonist promotes expression of PRDM16 in subcutaneous WAT [25,26,30,35,36]. It was reported that AMPK is essential for BAT development through the elevation of a key metabolite of the tricarboxylic acid cycle, a-ketoglutarate, which facilitates ten-eleven translocation mediated DNA demethylation of the PRDM16 gene promoter, and commits precursor cells to brown adipogenesis [25,27–29,37]. Our data indicate that inhibition of insulin signalling not only diminishes adipogenesis [16] but also represses human and mice white preadipocytes browning due to dysfunction of key factors in adipogenic differentiation. We reasoned that after inhibiting the insulin signalling, downregulation of PPARγ and C/EBPβ, is accompanied with an effect on the combination of PRDM16 with PPARγ, C/EBPβ, and PGC-1α, and finally inhibits UCP1 expression, so that it represses the preadipocytes transdifferentiation to beige adipocytes (Figure 7). Combining current and previous findings [16–19,22], it is clear that the insulin/insulin signalling is not only involved in white adipogenesis but also is invovled in regulating the expression of key browning molecules such as PRDM16, PGC-1α, and UCP1.

Based on the fact that a few numbers of white preadipocytes are still browning, we speculate that it is due to the insulin signalling being not completely blocked, has remained partially function, or other browning regulatory molecules are involved, such as insulin signalling independent and UCP-1 independent pathways [38,39]. Moreover, AMPK is an upstream regulatory activator of SITR1, which increases SITR1 activity by upregulating intracellular NAD^+^ levels, promotes the differentiation of brown preadipocytes, and causes white preadipocyte browning [27–29,37]. In the present study, pAMPK and SIRT1 increased significantly after incompletely suppressing insulin signalling, which compensated in part for the browning. This suggests that AMPK and SIRT1 are not only involved in the regulation of WACs browning, but are also involved in crosstalk with the insulin signalling. The cells from different donors show a similar tendency of beige adipogenesis, although they also showed slightly different sensitivities to browning stimuli and insulin signalling states, i.e. msASCs are more sensitive than that of hsASCs. It maybe a more sensitive defence response mechanism in the wild animal’s world, which is a development that has been conserved during long-term evolution to adapt to the stresses of the internal and external microenvironment, such as cold and nutrient deficiencies [1].

Overall, the insulin signalling positively regulates browning of white preadipocytes in human and mice, which not only promotes the process of adipogenesis, but also promotes the transcription and translation levels of brown molecules PRDM16, PGC-1α, and UCP1 [7,21,30,35,36]. These events occur by activating the insulin-PI3K-AKT-UCP1 pathway. After incompletely inhibiting the insulin signalling, it may participates in and promote a compensating browning through the AMPK-SIRT1 pathway [27]. Previous research has shown that non-obese and obese mouse primary adipose stem cells have different degrees of white adipogenic differentiation [40–43], while in the present study the tendency of expression of the above mentioned key factors in adipogenesis and browning-related factors seem to be consistent. This indicates that the phenomenon of preadipocyte browning commonly exist in different animal species such as humans and mice, as well as in different genotypes such as lean and obese animals, and the response to insulin signal is also similar although the degree is somehow different. SIRT1 deacetylates lysine residues of PGC-1α and PPARγ, promotes WACs browning, and improves insulin resistance [43]. However, such activation of AMPK-SIRT1 signal was not sufficient to fully induce beige adipogenesis when the insulin signalling was blocked, although the pathway is beneficial for the formation of beige cells [44]. It has been reported that disruption of insulin signalling in Myf5**^−^** derived brown adipocytes have reduced lipogenic gene expression in BAT and increased WACs browning [45,46], favouring insulin as the key AKT2 agonist driving de novo lipogenesis in BAT.

It has also been reported that cold exposure can increases proliferation, differentiation, and UCP1 activity, but also promotes the browning of WACs or adipocytes directly by sensimg temperature to activate thermogenesis [9,11,13,14,47], however the mechanism or relevance to white preadipocyte browning remains less understood. In our study, we conducted 26°C acclimation at different time periods during the browning of hsASCs and WT msASCs. The results clearly show that the period of low temperature (26°C) treatment is negatively correlated with the number and volume of lipid droplets, but is positively correlated with the degree of browning. This maybe because the temperature in the present study was lower than previously reported [11,14], and because the cells were exposed to 26°C in the middle and/or late stages of browning for 2 and/or 4 d. This suggests that although cold exposure increases thermogenesis in WACs, in temperatures colder than 32°C, it can also reduce lipid formation and accumulation, since this is the stage that is a crucial time for lipid accumulation and lipid droplet fusion. These characteristics indicate that the hsASCs and msASCs have dynamic phenotypic plasticity, and acquire a kind of cellular memory in the natural environment during evolution. Our results confirmed that primary white preadipocytes can be driven to form brown-like adipocytes in vitro by exposure to a low temperature, indicating that cold exposure can exert its effect on white primary preadipocytes directly in a cell-autonomous manner, activating a thermogenic programme that is independent of regulation of the central nervous system. However, when compared with WT or obese msASCs induced into white adipogenesis under 37°C, the lipid droplets were smaller and there was less adipogenesis when they were induced for beige adipogenesis under 37°C (Supplementary Figure S1).

## Conclusions

We provide evidence that the browning of human and mouse white primary preadipocytes was confirmed at the cell morphology, gene, and protein expression level using our modified method. Moreover, the results suggested that insulin signalling plays an important regulatory role in the process via PI3K-AKT-UCP1 signalling pathway. Dysfunction of the insulin signalling can significantly reduce white primary preadipocyte transdifferentiation into beige adipocyte. The effect of insulin on the process is independent of mammal species (humans and mice) of the cell donors, and mouse phenotype (lean and obese). In addition, the AMPK-SIRT1 signalling pathway is also involved in beige adipogenesis, although its ability to promote browning of white preadipocyte is limited. Moreover, cold acclimation reduces lipid production and accumulation in hsASCs and WT msASCs, while it promotes thermogenesis during beige formation (Figure 7). Other molecules independent of insulin signalling may also play a role in this process [38,39]. These questions offer fodder for further studies, and research deciphering these nutrients and internal and external environment sensing networks may offer new therapeutic targets to combat obesity and its related metabolic diseases.

## Data Availability

The authors confirm that the data supporting the findings of this study are available within the article [and/or] its supplementary materials.
